# Molecular Interplay of Brucellosis and Tuberculosis: Insights into Telomere Biology, Oxidative Stress, and Drug Resistance Mechanisms

**DOI:** 10.3390/diseases14070223

**Published:** 2026-06-23

**Authors:** Fatouma Mohamed Abdoul-Latif, Rohit Kumar, Yahya Ali Ismael, Houda Mohamed, Ali Merito, Saber Ali Ahmed, Reetu Yadav, Pannaga Pavan Jutur, Arpana Vibhuti

**Affiliations:** 1 Medicinal Research Institute, Center for Studies and Research of Djibouti (CERD), Djibouti City P.O. Box 486, Djibouti; soumade2015@gmail.com (Y.A.I.); abdoulhouda@yahoo.fr (H.M.); alimerito@gmail.com (A.M.); saberali32@yahoo.fr (S.A.A.); 2 Department of Biotechnology, Sri Ramaswamy Memorial Institute of Science and Technology, Delhi-NCR, Sonipat 131029, India; kumarrohitt121998@gmail.com; 3 Ganesh Scientific Research Foundation, Kirti Nagar, Delhi 110015, India; reetuyadav26197@gmail.com; 4 Centre Hospitalier Universitaire (CHU) of Djibouti, Djibouti City P.O. Box 2123, Djibouti; 5 International Centre for Genetic Engineering and Bio-Technology, Aruna Asaf Ali Marg, New Delhi 110067, India; pavan.jutur@icgeb.org

**Keywords:** brucellosis, tuberculosis, telomere biology, drug resistance, ROS, immune senescence

## Abstract

Brucellosis and tuberculosis (TB) are chronic infectious diseases of international public health importance, with developing countries being most affected. The diagnosis of brucellosis and tuberculosis co-infection remains challenging because both diseases present with overlapping nonspecific clinical manifestations, such as prolonged fever, fatigue, and weight loss, and elicit similar cell-mediated immune and inflammatory responses, which can complicate differential diagnosis, particularly in endemic regions. Recently, it has been shown that chronic infections affect cell stress pathways such as oxidative stress and telomere function. The current literature review provides an overview of the relationship between brucellosis and TB at a molecular level, focusing on telomere biology, oxidative stress and the mechanisms of antimicrobial resistance. Due to chronic immune response in brucellosis and TB patients, an increase in reactive oxygen species (ROS) levels is observed, leading to DNA damage and subsequent telomere shortening and alteration of telomerase activity. These alterations might be responsible for immune senescence, weakened defense response and persistent infection. In addition, different methods of drug resistance have been discovered among brucellae and mycobacteria, such as mutation in target sites, efflux systems and intracellular persistence, making their eradication difficult. Finally, the potential role of telomere-related genes and biomarkers of oxidative stress in diagnosis and prognosis is also highlighted. Insights into these interrelated pathways would allow us to have a better understanding of host–pathogen interactions and hence offer a possible means of developing new strategies in the fight against co-infection by finding new biomarkers.

## 1. Introduction

Brucellosis and tuberculosis (TB) remain formidable challenges to global public health, particularly in low- and middle-income countries where they are endemic [1,2]. In combination, all these bacterial diseases affect a huge number of people each year, causing morbidity, economic losses, and even death. Brucellosis, a zoonotic infection, which is caused by bacteria from the *Brucella* genus, especially *B. melitensis*, *B. abortus*, and *B. suis*, is contracted in humans from infected animals, contaminated unpasteurized milk, or inhalation of aerosols [3]. Tuberculosis, caused by *Mycobacterium tuberculosis*, spreads via airborne droplets and primarily affects the lungs, though it can disseminate to extrapulmonary sites. Despite their different etiological origins, both pathogens share a critical pathogenic feature: they are facultative intracellular organisms that have evolved sophisticated mechanisms to survive and replicate within host immune cells, particularly macrophages [3,4]. This intracellular lifestyle enables them to evade host immune responses, establish persistent infections, and cause chronic disease that can last for years if untreated.

There is significant geographic association between brucellosis and tuberculosis, particularly within the Mediterranean region, the Middle East, certain parts of Asia, Central/South America, and sub-Saharan Africa. Within this geographical area, livestock rearing is practiced, tuberculosis rates are high, and health care facilities can be few and far between. There is a significant geographic overlap between brucellosis and tuberculosis, particularly in the Mediterranean basin, the Middle East, parts of Asia, and sub-Saharan Africa, where both diseases remain endemic. Although documented *Brucella–Mycobacterium tuberculosis* co-infection appears to be relatively rare, published case series and epidemiological reports from co-endemic regions suggest that its prevalence is likely underestimated because of overlapping clinical features and limited routine testing for both pathogens [5,6].

The main reason for this diagnostic discrepancy is the striking resemblance in the presentation of both disorders. Both conditions usually manifest themselves through a range of symptoms such as fever, sweating at night, extreme tiredness, unexpected weight loss, joint pains, and loss of appetite [7]. As far as brucellosis is concerned, hepatosplenomegaly and lymphadenopathy are also some symptoms of this infection, whereas TB can cause symptoms such as chronic cough, hemoptysis, and chest pain if it affects the lungs of a patient. When both these infections happen simultaneously, they can create oxidative stress in cells, leading to DNA damage with telomere depletion. They also create further confusion regarding the proper diagnosis of patients, since they show many overlapping symptoms. A patient may be diagnosed and treated with the wrong type of disease and antibiotics due to this problem [8]. In endemic regions, clinical algorithms that rely solely on symptom assessment frequently fail to distinguish between these entities, underscoring the urgent need for better molecular and biomarker-based diagnostic tools [9].

On a cellular level, both brucellosis and tuberculosis are marked by a sustained immune response. Following the infection, the host cells, including macrophages and dendritic cells, recognize the pathogen-associated molecular patterns (PAMPs) with the help of the pattern recognition receptors (PRRs) like Toll-like receptors (TLRs) [10]. It leads to a series of pro-inflammatory signal transduction that involve the nuclear factor kappa B (NF-κB) and mitogen-activated protein kinases (MAPKs). This results in the secretion of various cytokines, including tumour necrosis factor alpha (TNF-α) and interleukin 1beta (IL-1β) [11]. In addition, reactive oxygen species (ROS) and reactive nitrogen species (RNS) are produced by the activation of phagocytes through the activity of nicotinamide adenine dinucleotide phosphate (NADPH) oxidase complex and inducible nitric oxide synthase (iNOS) enzymes, respectively. These chemicals are designed to eliminate or inhibit the survival of pathogens within the cell [12]. In chronic infection cases, however, this defense strategy can be viewed as a two-sided weapon. The prolonged activity of the immune response results in constant ROS and RNS generation, outpacing the body’s natural defenses against antioxidants. This situation is called oxidative stress, which results in collateral damage to the body’s own components, such as protein (carbonylation, nitration), lipids (lipid peroxidation), and DNA (strand breakage, base alterations) [13]. Among the most susceptible segments of genomic DNA are telomeres, composed of TTAGGG nucleotide repeats that form the chromosome ends. Telomeres are highly reactive towards oxidants due to their large amount of guanine residues, which can be easily oxidized to form 8-oxo-7,8-dihydroguanine (8-oxo-dG). Oxidative damage to telomeres causes telomere shortening and activates the DNA damage response, leading to genomic instability, which is also discussed in Table 1 [14].

The biology of telomeres is a new but highly important area that needs to be addressed for chronic infections. Telomeres help guard chromosomes against fusing together, being degraded, and engaging in illegitimate recombination. Telomeres are largely stabilized by telomerase, which is a ribonucleoprotein enzyme consisting of a telomerase reverse transcriptase (TERT) subunit and a telomerase RNA component (TERC) [25]. The telomere length decreases with each replication process in healthy subjects and acts as a cellular replicative life span meter. However, in the case of oxidative stress and inflammation, this process becomes much faster. In relation to tuberculosis infection, there is evidence that shows that in peripheral blood mononuclear cells (PBMCs) of the infected subjects, the telomeres are shorter than those in the healthy subjects [26]. While some studies show that successful therapy against TB disease could help recover telomere length, brucellosis remains poorly understood in this regard. Notably, no specific study has evaluated the role of telomeres in brucellosis infection in humans, nor has there been any investigation of telomeres in patients with concurrent infections by *Brucella* spp. and *M. tuberculosis* [27]. This knowledge gap is striking, given the chronic nature of brucellosis, its capacity to induce prolonged oxidative stress, and its epidemiological overlap with TB.

Yet another dimension of complexity is posed by the increasing issue of drug resistance. Multidrug-resistant TB (MDR-TB) refers to TB that refers to tuberculosis that is resistant to multiple drugs, such as isoniazid and rifampicin. MDR-TB poses a huge challenge to global health, with half a million new cases occurring each year [28]. XDR-TB involves the addition of fluoroquinolones and second-line injectable antibiotics. With regard to brucellosis, although acquired resistance is less likely to occur, there is frequent failure and relapses of the disease due to the persistence of bacteria intracellularly, the failure of antibiotics to penetrate into the bacteria, or insufficient duration of the treatment course. Relapse rates with brucellosis treatment can range from 5% to 15% despite combination therapy with appropriate drugs such as doxycycline in conjunction with either rifampicin or an aminoglycoside. Notably, rifampicin, which is commonly used in the treatment of TB, is also used in the treatment of brucellosis. The extensive use of rifampicin in treating TB in endemic regions is likely to lead to the selection of rifampicin-resistant *Brucella* species, although this aspect has not been systematically investigated [15]. Understanding the molecular mechanisms that drive drug resistance in both pathogens and how these mechanisms intersect with oxidative stress and telomere dysfunction is therefore an urgent research priority.

This review aims to compile the current knowledge on the molecular interplay between brucellosis and TB, with a specific focus on three interconnected domains based on the role of oxidative stress in disease pathogenesis and host damage; the impact of chronic infection on telomere biology and cellular ageing; and the mechanisms of drug resistance in each infection and how co-infection may exacerbate treatment challenges. Through this combination of themes, it is hoped that a complete theoretical framework will be developed that will guide future research, suggest possible biomarkers for diagnostic purposes, and indicate new treatment options for patients afflicted with these debilitating chronic infections.

## 2. Pathogenesis of Brucellosis and Tuberculosis

### 2.1. Brucellosis Pathogenesis

The genus *Brucella* consists of Gram-negative bacteria with the ability to be a facultative intracellular organism with an extremely evolved mechanism of subverting the host immune system. Entry usually occurs through the mucosa of the oropharynx, gastrointestinal tract, and respiratory tract.

**Figure 1:**
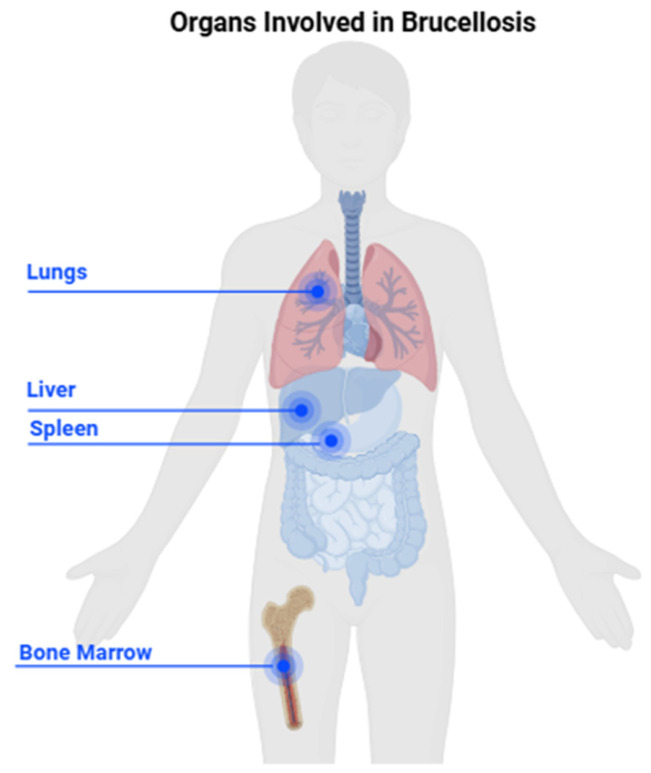
The schematic diagram showing the organs that are primarily affected by brucellosis. It can be noted from the schematic illustration that brucellosis affects various body organs. Among them are bone marrow, lungs, liver, and spleen. Infection of the bone marrow is linked to the occurrence of such disorders as spondylitis, which primarily targets the spinal cord. The lungs constitute a relatively uncommon site for the infection but are still considered clinically important in cases of brucellosis. The liver and spleen infections may present as hepatosplenomegaly. Created in BioRender. Rohit Kumar (2025). https://www.biorender.com/, accessed on 1 May 2026.

As illustrated in Figure 1, the organs involved in brucellosis [29]. Including the skin, lungs, mouth, lymph nodes, spleen, liver, gastrointestinal tract, bone marrow, and genital tract. The next step involves the uptake of the *Brucella* organisms by the professional phagocytic cells, such as macrophages and dendritic cells. Unlike other intracellular bacteria [30]. The bacteria release type IV secretion system (T4SS) proteins, which are coded for by the virB operon. The bacterial effector proteins that are released by the bacterium target the vesicle traffic, thus blocking the development of the phagosome into a lysosome. Rather than developing into a lysosome, the Brucella-containing vacuole (BCV) acquires the membrane markers of the endoplasmic reticulum (ER) and later converts into an ER-like niche [31]. In this enclosed environment, the *Brucella* species multiplies without causing inflammatory stimuli. The pathogen also suppresses the host’s immune system through inhibiting TLR signaling, decreased levels of TNF-α production, and induction of an anti-inflammatory state where IL-10 prevails [32]. This mode of immune escape makes *Brucella* capable of developing chronic and long-lasting infections. One of the most prominent pathological features of chronic brucellosis is the development of granulomas. These are defined as organized collections of macrophages, lymphocytes, and multinucleated giant cells. Nevertheless, the granulomas formed in brucellosis cases are not caseating granulomas like those seen in tuberculosis cases and can be dissolved independently [33].

### 2.2. Tuberculosis Pathogenesis

*Mycobacterium tuberculosis* is an acid-fast, slow-growing bacillus with a unique lipid-rich cell wall that contributes to its virulence and drug resistance. Infection begins when aerosolized droplets containing the bacteria are inhaled and reach the alveolar spaces. By maintaining the phagosome in an early endosomal state, *M. tuberculosis* avoids fusion with lysosomes, thereby escaping exposure to acidic pH, hydrolytic enzymes, antimicrobial peptides, and high concentrations of reactive oxygen and nitrogen species. At the same time, this compartment remains metabolically active and nutrient-accessible, enabling intracellular bacterial survival and replication [34]. Alveolar macrophages are the first line of defence, engulfing the bacilli through phagocytosis. However, *M. tuberculosis* has evolved multiple strategies to survive within these hostile cells. The bacterium produces a variety of factors that inhibit phagosome-lysosome fusion, including sulfolipids, trehalose dimycolate (cord factor), and the secreted protein kinase G (PknG) [35]. It also prevents phagosomal acidification by excluding the vacuolar ATPase (V-ATPase) proton pump from associating with the macrophage phagosomal membrane, particularly through interference with the recruitment of the V-ATPase subunit H, thereby preventing luminal acidification and lysosomal maturation [36]. The *M. tuberculosis*-containing phagosome retains an early endosomal phenotype, allowing the bacterium to access nutrients and avoid degradation [37]. Like *Brucella*, *M. tuberculosis* manipulates host cell death pathways, inhibiting apoptosis while promoting necrotic cell death, which facilitates bacterial dissemination. The hallmark of TB pathology is the granuloma, a highly organized immune structure that contains the infection but also serves as a site of bacterial persistence [38]. Within the granuloma, *M. tuberculosis* can enter a dormant, non-replicating state characterized by altered metabolism, reduced cell wall synthesis, and increased tolerance to antibiotics. Reactivation of dormant bacteria is a major cause of active TB in latently infected individuals, especially when immunity is compromised by conditions such as human immunodeficiency virus (HIV) co-infection, diabetes mellitus, malnutrition, or ageing, all of which impair protective cellular immune responses and increase the risk of latent TB reactivation [39,40].

### 2.3. Co-Infection Dynamics

At present, there is insufficient evidence to conclude that either pathogen consistently precedes the other. Rather than representing a strictly simultaneous process, co-infection may occur sequentially, whereby chronic immune dysregulation induced by one pathogen facilitates establishment or reactivation of the other. For example, latent tuberculosis-associated immune exhaustion and granulomatous remodeling could reduce host capacity to eliminate intracellular *Brucella*, whereas persistent *Brucella* infection and IL-10-mediated immunomodulation may weaken Th1-mediated antimicrobial responses, potentially favoring reactivation of latent *M. tuberculosis*. Thus, the interaction is likely bidirectional and context-dependent rather than universally synergistic. The presence of both *Brucella* and *M. tuberculosis* in the same organism is not just additive but synergistic for causing an immune imbalance. Both microorganisms have a tendency to infect similar intracellular locations such as macrophages and cause immune reactions that produce interferon (IFN)-γ, activate macrophages, and form granulomas [41].

**Figure 2:**
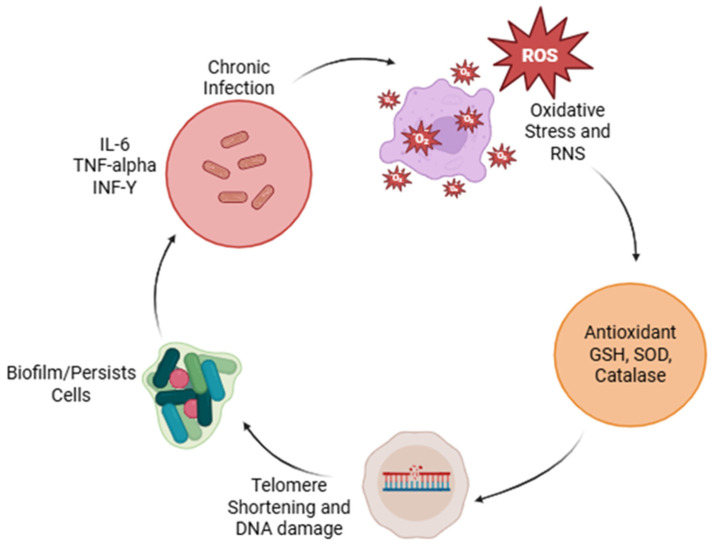
A schematic diagram depicting the interaction between chronic infections, oxidative stress, and telomere shortening. Chronic infection causes prolonged production of pro-inflammatory cytokines, such as IL-6, TNF-α, and IFN-γ, which contribute to the production of ROS and RNS, causing oxidative stress. The increased oxidative stress is a challenge for the cellular antioxidant defense mechanisms, such as GSH, SOD, and catalase. Oxidative stress causes shortening of telomeres and damage to DNA, leading to compromised cell structure. Cell damage and telomere shortening aid in the establishment of biofilm or resistant bacteria, leading to chronic infection and a vicious cycle of pathology. Abbreviations: GSH, glutathione; IFN, interferon; IL, interleukin; ROS, reactive oxygen species; RNS, reactive nitrogen species; SOD, superoxide dismutase; TNF, tumor necrosis factor. Created in BioRender. Rohit Kumar (2025). https://www.biorender.com/, accessed on 1 May 2026.

T-cell exhaustion due to prolonged antigenic stimulation can occur in patients suffering from both infections; this may be accompanied by increased expression of inhibitory receptors including programmed cell death-1 (PD-1), cytotoxic T-lymphocyte antigen-4 (CTLA-4), and T-cell immunoglobulin and mucin-domain containing-3 (TIM-3), as well as reduced production of effector cytokines. This will prevent the body from clearing the infection [42]. Prolonged antigenic stimulation during chronic *Brucella* and *M. tuberculosis* infection may induce a state of T-cell exhaustion or functional dysregulation, characterized by sustained expression of inhibitory receptors including PD-1, CTLA-4, TIM-3, and lymphocyte activation gene-3 (LAG-3), impaired T-cell receptor signaling, altered metabolic programming, and reduced production of effector cytokines such as IFN-γ and TNF-α. Although the term ‘T-cell exhaustion’ is widely used, the immune alterations observed in chronic bacterial infections may represent a spectrum ranging from reversible functional dysregulation and anergy to terminal exhaustion [43]. Furthermore, the continuous state of inflammation results in higher levels of oxidative stress compared to cases of isolated infection Figure 2. It is still unknown what kind of competition for nutrients might occur or whether bacteria can interact directly or indirectly.

### 2.4. Oxidative Stress in Brucellosis and TB

In oxidative stress, which is an essential pathophysiologic component of brucellosis and TB, there is an interaction between chronic inflammatory response, tissue injury, immunological malfunction, and genetic instability.

In healthy individuals, there is an equilibrium of free radical generation and neutralization. However, this balance becomes unbalanced in chronic infections [44].

In TB, there is ample evidence of increased oxidative stress biomarkers. The plasma concentration of malondialdehyde (MDA), which results from lipid peroxidation, is consistently higher among TB-infected subjects relative to healthy individuals. It also falls following effective therapy of the disease [45]. In the same way, high levels of 8-oxo-dG from urine and plasma, an indicator of DNA oxidative damage, have been found in TB cases, and these levels correlate positively with disease severity and bacterial burden. TB patients have decreased antioxidative status in the form of decreased levels of glutathione (GSH), total antioxidant status (TAS), and decreased enzyme activities like superoxide dismutase (SOD), catalase (CAT), and glutathione peroxidase (GPx). In some studies, the ratio of oxidized glutathione (GSSG) to glutathione (GSH) was found to be significantly higher [46]. It should be noted that the bacterium itself is subjected to reactive oxygen species produced by the host and, hence, has developed its own antioxidant mechanisms comprising catalase-peroxidase (KatG), superoxide dismutase (SodA, SodC), and alkyl hydroperoxide reductase (AhpC). The role of these antioxidant enzymes in protecting the bacteria is supplemented by their ability to regulate the redox state of the host. Catalase-peroxidase (KatG), for instance, is involved in the activation of the prodrug isoniazid; katG gene mutations have been identified as the principal cause of isoniazid resistance [47].

However, the pattern of oxidative stress in cases of brucellosis is not that well-researched. It was found out that in patients with both acute and chronic forms of the disease, serum MDA levels are elevated, and antioxidant enzyme activity (SOD, GPx) is diminished [48]. Nitric oxide (NO) overproduction has been observed in some other studies, evidenced by nitrite/nitrate accumulation. The genus *Brucella* is endowed with several antioxidants, such as SODs (SodA, SodC), catalase (KatE), and an unusual periplasmic cytochrome c peroxidase (Ccp). This helps *Brucella* to survive oxidative damage during the burst response of activated macrophages, leading to chronic infection. Notably, the genus *Brucella* does not have a KatG orthologue; therefore, it is intrinsically more sensitive than *Mycobacterium tuberculosis* to oxidative conditions. This differential antioxidant capacity may also influence the competition and coexistence dynamics of both pathogens within co-infected macrophages, potentially making *Brucella* more reliant on the host oxidative stress milieu for intracellular survival and persistence [49].

It would seem logical to postulate that there should be an enhancement of oxidative stress in co-infected patients because the burden of two organisms within the same cell will stimulate increased ROS and RNS formation along with depleting the body’s supply of antioxidants. As a result, there would be faster degradation of DNA, loss of telomeres, and premature immune senescence. However, there is still no data on co-infections available [50].

### 2.5. Telomere Biology and Chronic Infection

Telomeres are made up of tandem repeats of TTAGGG nucleotides along with shelterin protein complex TRF1, TRF2, POT1, TIN2, TPP1, and RAP1. Their main biological role is to recognize the true chromosome ends and protect them from being repaired as broken DNA molecules [51]. Telomere length decreases during each round of cell division owing to the end replication problem and oxidative damage to DNA. Oxidized guanines that accumulate at the telomere sequence are not efficiently removed via base excision repair pathways. Instead, these lesions result in DNA breaks, which accelerate the loss of telomere length. Short telomeres result in senescence and apoptosis of immune cells [52].

Telomere shortening has been shown to occur due to chronic infections such as TB. TB patients were found to have shorter telomere length in PBMCs compared to control individuals, and telomere shortening was positively related to high bacterial load and radiologic disease severity [53]. Other research discovered that the level of telomerase expression was decreased in T-cells from patients with tuberculosis, and thus telomeres were not maintained properly. However, once patients were cured of their diseases, there was evidence that there was a partial restoration of telomere length. In cases of co-infection of HIV and tuberculosis, telomere shortening became even more obvious [54].

For brucellosis, no direct studies evaluating telomere length or telomerase activity have been published to date. However, considering that chronic brucellosis induces persistent inflammation and oxidative stress, an important question is whether it may promote telomere shortening and immune cell ageing like, or potentially more severe than, tuberculosis, particularly under co-infection conditions where cumulative oxidative and inflammatory burden may be amplified [53]. In co-infected patients, the combined effect of two chronic bacterial infections, each driving immune cell proliferation and oxidative damage, could lead to profound telomere shortening, accelerating immune senescence.

This, in turn, would impair the host’s ability to control both infections, creating a vicious cycle of persistent inflammation, immune exhaustion, and increasing pathogen burden [55].

Telomere-associated proteins also warrant attention. TRF2, for example, protects telomeres from ATM kinase-mediated DNA damage responses and is highly sensitive to oxidative modification. Chronic oxidative stress could impair TRF2 function, leading to telomere deprotection and genomic instability even in the absence of extreme telomere shortening [56]. Similarly, POT1, which binds single-stranded telomeric overhangs, can be inactivated by ROS. Future studies should investigate not only telomere length but also the integrity of the shelterin complex in TB and brucellosis patients [57].

## 3. Drug Resistance Mechanisms

### 3.1. Drug Resistance in Tuberculosis

Resistance to tuberculosis drugs occurs genetically, as a result of spontaneous mutations in genes on the chromosome. In the case of rifampicin, the strongest anti-TB drug, the cause for resistance occurs due to mutations in the gene rpoB, responsible for encoding the β subunit of RNA polymerase [58]. More than 95% of rifampicin-resistant isolates have mutations within an 81-base pair segment in *rpoB* known as the rifampicin resistance-determining region (RRDR). Such mutations include Ser531Leu and His526Asp, which decrease rifampicin binding [59]. Isoniazid resistance requires several genetic modifications and is complicated because it is mediated by multiple mutations. The most common type of katG mutation leads to elimination or reduced catalase-peroxidase activity, which results in the failure of isoniazid activation. Mutation in the promoter region of inhA leads to overproduction of its protein target (enoyl-ACP reductase) and consequently results in isoniazid resistance. Other rare mutations occur in ahpC, kasA, and ndh [60]. Fluoroquinolone resistance, seen in MDR-TB treatment, results from mutations in the quinolone resistance determining region (QRDR) in gyrA and gyrB genes encoding the DNA gyrase enzyme. The second-line injectables such as amikacin, kanamycin, and capreomycin get deactivated due to mutations in the rrs gene (encoding for 16S rRNA) and eis promoters. Apart from genetic mutations in TB bacteria, there are efflux pumps (like Rv1258c, Rv1410c), which pump out the drug and help in achieving resistance to these drugs at low levels while developing high-level resistance. Unlike biofilms in other bacteria, biofilms in *M. tuberculosis* are not very common [61].

### 3.2. Drug Resistance in Brucellosis

The *Brucella* species exhibit natural resistance to multiple drugs like colistin, bacitracin, and various β-lactams, owing to their non-permeability and use of efflux pumps. However, for the combination therapy using doxycycline and rifampicin or doxycycline and an aminoglycoside like streptomycin or gentamicin, the occurrence of resistance is very rare but still exists [62]. *Brucella’s* resistance to rifampicin involves mutations within rpoB, just like TB bacteria, thereby creating the potential that antibiotic-resistance programs against TB could lead to rifampicin-resistant strains of Brucella [63]. Resistance to doxycycline is uncommon but can be due to efflux pumps like TetA and TetB or mutations in the 16S rRNA genes. Resistance to aminoglycosides can be achieved by aminoglycoside-modifying enzymes like AAC(6′)-I and APH(3′)-I or mutations in the ribosomal proteins. The biggest threat faced by clinicians is not resistance but relapse [64]. *Brucella* is capable of surviving intracellularly for extended periods, avoiding any contact with antibiotics, only to resurface when treatment is discontinued. Such a phenomenon does not represent resistance, but persistence or tolerance, potentially associated with the existence of non-dividing persister cells, characterized by metabolic dormancy and higher survival rates in the presence of antibiotics. The formation of such cells can potentially be stimulated by oxidative stress in both *Brucella* and *M. tuberculosis* [65].

### 3.3. Impact of Co-Infection on Drug Resistance

Co-infection with *Brucella* and *M. tuberculosis* complicates drug therapy in several ways. First, treatment regimens overlap: rifampicin is used in both standard TB therapy (Rifampicin, Isoniazid, Pyrazinamide, Ethambutol; RHZE) and standard brucellosis therapy (doxycycline plus rifampicin) [8]. A co-infected patient may therefore receive prolonged rifampicin therapy, increasing the risk of rifampicin resistance in either pathogen. Second, drug–drug interactions are possible, particularly involving rifampicin, a potent inducer of cytochrome P450 enzymes. Rifampicin can reduce serum levels of doxycycline, potentially compromising brucellosis treatment [66]. Third, the immune dysregulation and oxidative stress in co-infection may impair the efficacy of antibiotics that require host immune cooperation, such as isoniazid, which is a prodrug whose bactericidal activity depends on activation by the mycobacterial catalase-peroxidase enzyme KatG. Mutations or functional impairment of KatG reduce drug activation and are a principal mechanism of isoniazid resistance. In addition, intracellular redox conditions may influence the efficacy of other antimicrobials, including pyrazinamide and nitroimidazole derivatives, whose activity is linked to bacterial metabolic state and oxidative balance [67]. Fourth, co-infection may promote the selection of multidrug-resistant strains by creating a high-burden, prolonged, and suboptimally treated environment. Systematic studies of drug resistance patterns in co-infected patients are urgently needed [68].

Standard first-line tuberculosis treatment includes rifampicin administration for at least six months, whereas uncomplicated brucellosis is commonly treated with doxycycline plus rifampicin for approximately six weeks. Consequently, patients with co-infection may experience extended cumulative rifampicin exposure, potentially increasing the selective pressure for emergence of rifampicin-resistant mutants. Experimental and clinical studies have demonstrated that prolonged or suboptimal rifampicin exposure promotes the selection of resistant rpoB mutant populations [69]. Direct evidence linking *Brucella–M. tuberculosis* co-infection with the emergence of multidrug resistance remains limited. Nevertheless, published case reports describing co-infected patients have highlighted prolonged treatment courses, diagnostic delays, and repeated antibiotic exposure, all of which may create conditions favorable for the selection of resistant strains. The absence of systematic surveillance studies represents a major knowledge gap and warrants future multicenter investigations [70].

### 3.4. Interconnection Between Telomere Biology, Oxidative Stress, and Drug Resistance

The three topics discussed above have an intrinsic relationship and are interconnected. Firstly, oxidative stress leads to direct damage to the structure of telomeres, resulting in their rapid degradation. Secondly, dysfunctional and shortened telomeres contribute to cellular senescence and exhaustion of the host’s immune response, preventing the elimination of intracellular pathogenic microorganisms. Finally, persistent infection causes oxidative stress [71]. In this cycle, drug resistance can occur or be enhanced. Mutation rates can be increased by DNA damage caused by oxidative stress. ROS in *M. tuberculosis* can induce oxidative DNA lesions, which can facilitate faster development of drug resistance-conferring mutations [72]. On the contrary, successful pharmacotherapy decreases bacterial load and inflammation, leading to reduced oxidative stress and telomere repair. Thus, telomere length as well as oxidative stress parameters can be used as biomarkers of response to pharmacotherapy [63]. Antioxidants like N-acetylcysteine (NAC) or vitamin C can decrease oxidative stress, increase efficiency of antibiotics and prevent telomere shortening. However, no studies on their efficacy in clinical practice have been conducted so far. Finally, modulation of telomerase activity (such as TERT activators) represents an innovative strategy to promote immunity [73].

## 4. Clinical Symptoms and Implications with Future Perspectives

**Figure 3:**
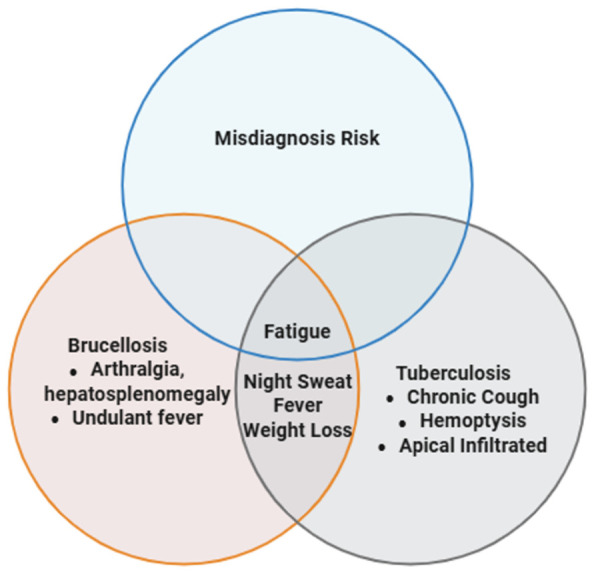
Venn diagram illustrating the overlap and distinctness in clinical manifestations of brucellosis and tuberculosis, along with the associated risk of misdiagnosis. Brucellosis (orange circle) is characterized by arthralgia, hepatosplenomegaly, and undulant fever, whereas tuberculosis (grey circle) presents with chronic cough, hemoptysis, and apical infiltrates. The overlapping region highlights shared symptoms, including fever, night sweats, weight loss, and fatigue, which contribute significantly to diagnostic challenges. The upper intersecting area emphasizes the increased risk of misdiagnosis due to these nonspecific and overlapping clinical features, particularly in endemic settings. The overlapping regions highlight shared symptoms, including fever, night sweats, weight loss, and fatigue, which contribute to an increased risk of misdiagnosis and delayed diagnosis, particularly in endemic settings. Created in BioRender. Rohit Kumar (2025). https://www.biorender.com/, accessed on 1 May 2026.

Both brucellosis and TB have overlapping symptoms, which are depicted in Figure 3, such as fever, malaise, and weight loss, which can be difficult to distinguish from each other. In areas where TB is endemic, health care professionals need to be on the lookout for cases of brucellosis in patients who have been in contact with animals or consumed unpasteurized milk [74]. The presence of persistent fever, arthralgia, or hepatosplenomegaly in patients undergoing anti-tuberculosis therapy should prompt consideration of concurrent *Brucella* infection and appropriate serological testing [75].

From a clinical perspective, new molecular biomarkers like telomere length, telomerase expression, and oxidative stress, including MDA, 8-oxo-dG, and the GSH/GSSG ratio, have promising potential in disease staging, risk of relapse prediction, and drug efficacy evaluation [26]. However, their application remains largely investigational, and robust validation through large-scale, longitudinal studies is required before routine clinical implementation.

Adjunct therapy that focuses on oxidative stress could be beneficial from a therapeutic point of view. Antioxidants like N-acetylcysteine and vitamin C have been suggested for reducing oxidative stress, which may improve immune response and increase antimicrobial effectiveness [76]. However, there is little proof to support this, and clinical studies are required to determine their effectiveness. Future work must focus on conducting exhaustive epidemiological studies that will help determine the true prevalence of the disease. At the same time, efforts should be made to conduct mechanistic studies that can help shed light on the involvement of oxidative stress and telomere dynamics in the disease process. It is just as important to conduct clinical trials focusing on the effects of treatment in co-infected patients, as well as issues of drug resistance and host–pathogen interactions.

The growing burden of antimicrobial resistance necessitates the development of innovative therapeutic strategies beyond conventional antibiotic regimens. One promising approach involves ‘Trojan Horse’ antibiotics, which exploit bacterial nutrient acquisition systems to facilitate intracellular drug delivery and overcome permeability barriers associated with resistant pathogens [77]. Likewise, targeting bacterial metallophore-mediated metal acquisition pathways represents an attractive anti-virulence strategy because these systems are essential for pathogen survival, persistence, and adaptation within the host [78]. In parallel, bacteriophage-based therapies and phage-derived antimicrobial products have emerged as potential alternatives or adjuncts to conventional antibiotics, particularly against multidrug-resistant bacterial infections. Future studies integrating these advanced antimicrobial technologies with host-directed approaches aimed at restoring redox balance and preserving telomere integrity may provide novel and more effective therapeutic strategies for the management of brucellosis, tuberculosis, and their co-infections [79].

Beyond improving mechanistic understanding, the molecular pathways discussed in this review have significant translational potential. Biomarkers associated with oxidative stress and telomere biology, including telomere length, telomerase activity, MDA, 8-oxo-dG, and GSH/GSSG ratio, may serve as complementary tools for disease staging, prediction of relapse, monitoring of therapeutic response, and early recognition of *Brucella–Mycobacterium tuberculosis* co-infection in endemic settings. Combining these host-derived biomarkers with conventional microbiological and molecular diagnostic methods could improve diagnostic accuracy and reduce delays in initiating appropriate therapy [80].

The pathways described herein also suggest several potential host-directed therapeutic approaches. Adjunctive antioxidant therapies, such as N-acetylcysteine or vitamin C, may help restore redox homeostasis, reduce oxidative tissue damage, and potentially enhance antimicrobial efficacy. Likewise, interventions targeting telomere maintenance and cellular senescence pathways may represent innovative strategies for preserving immune competence during chronic intracellular infections, although these approaches require extensive preclinical and clinical validation [81].

To facilitate translation of these concepts into clinical practice, robust experimental models are needed. Animal models of *Brucella–M. tuberculosis* co-infection, particularly murine systems that recapitulate chronic intracellular persistence and immune dysregulation, could provide valuable platforms for investigating disease pathogenesis, validating biomarker candidates, and evaluating novel host-directed therapies before clinical application. Such integrated experimental and clinical approaches may ultimately contribute to the development of personalized diagnostic and therapeutic strategies for patients with dual intracellular bacterial infections [82].

## 5. Conclusions

*Brucellosis* and *M. tuberculosis* co-infection represents a complex, understudied, and clinically challenging intersection of two chronic intracellular bacterial infections. Chronic inflammation and sustained production of reactive oxygen and nitrogen species lead to oxidative stress, causing collateral damage to host tissues, including accelerated telomere shortening and genomic instability. Telomere attrition, in turn, promotes immune senescence and reduces the host’s capacity to eliminate pathogens, perpetuating a vicious cycle of infection, inflammation, and tissue damage. Drug resistance, already a major problem in TB and an emerging concern in brucellosis, is likely exacerbated by co-infection through overlapping treatment regimens, drug–drug interactions, and oxidative stress-induced mutagenesis. Understanding the molecular interplay among oxidative stress, telomere biology, and drug resistance is not only scientifically fascinating but also clinically urgent. It offers the promise of novel biomarkers for diagnosis and prognosis, as well as new therapeutic avenues that target not only the pathogens themselves but also the host’s redox and telomere maintenance pathways. Future research should prioritize well-designed clinical studies of co-infected patients, animal models that recapitulate dual infection, and mechanistic investigations at the host–pathogen interface. Only through such integrated efforts can we hope to improve outcomes for the millions of people living in regions where brucellosis and tuberculosis continue to exact a heavy toll.

## Figures and Tables

**Table 1:** Comparative overview of brucellosis and tuberculosis pathogenesis, oxidative stress, telomere biology, drug resistance mechanisms, and clinical features, with emphasis on the impact of co-infection. ↑ indicates increased levels, activity, expression, or abundance; ↓ indicates decreased levels, activity, expression, or abundance relative to healthy controls or baseline conditions. Abbreviations: ER, endoplasmic reticulum; GPx, glutathione peroxidase; GSH, glutathione; IFN, interferon; IL, interleukin; MDA, malondialdehyde; 8-oxo-dG, 8-hydroxy-2′-deoxyguanosine; SOD, superoxide dismutase; TNF, tumour necrosis factor.

Feature	Brucellosis	Tuberculosis	Impact of Co-Infection	References
Causative Agent	*Brucella melitensis*, *B. abortus*, *B. suis* (Gram-negative coccobacillus)	*Mycobacterium tuberculosis* (acid-fast bacillus)	Dual intracellular infection: potential competition for macrophage niches	[15]
Primary Reservoir	Domestic animals (goats, cattle, swine); zoonotic	Humans (airborne transmission)	Overlap in endemic rural/agricultural regions	[16]
Intracellular Niche	Brucella-containing vacuole (BCV) that acquires ER markers	*M. tuberculosis*-containing phagosome that inhibits lysosomal fusion	Both evade phagolysosome fusion; may compete for host resources	[17]
Immune Response	Th1-mediated (IFN-γ, TNF-α) but with anti-inflammatory IL-10 modulation	Th1-dominated (IFN-γ, IL-12, TNF-α); granuloma formation	Potential immune exhaustion and dysregulation	[18]
Oxidative Stress Markers	↑ MDA, ↓ SOD, ↓ GPx; limited human studies	↑ MDA, ↑ 8-oxo-dG, ↓ GSH, ↓ total antioxidant capacity	Likely amplified oxidative stress; more severe DNA damage	[19]
Telomere Biology	Telomere shortening	Shorter telomere length in PBMCs; altered telomerase activity	Expected accelerated telomere attrition and immune senescence	[20]
Primary Drug Resistance Mechanisms	*rpoB* mutations (rifampicin); Efflux pumps (TetA/B for doxycycline); Intracellular persistence/relapse	*rpoB* mutations (rifampicin); *katG*/*inhA* mutations (isoniazid); *gyrA/B* (fluoroquinolones); Efflux pumps	Overlapping rifampicin use; risk of cross-resistance; drug–drug interactions (e.g., rifampicin reduces doxycycline levels)	[21]
Standard Treatment	Doxycycline + rifampicin OR doxycycline + gentamicin/streptomycin	RHZE (rifampicin, isoniazid, pyrazinamide, ethambutol)	Prolonged rifampicin exposure; complex regimens; higher relapse risk	[22]
Common Clinical Overlap	Fever, night sweats, weight loss, fatigue, arthralgia, hepatosplenomegaly	Fever, night sweats, weight loss, fatigue, chronic cough, hemoptysis	High risk of misdiagnosis or delayed treatment	[23]
Pathognomonic Finding	Non-caseating granulomas (liver, spleen, bone marrow)	Caseating granulomas with Langhans giant cells (lungs, lymph nodes)	Histology alone cannot distinguish; requires culture or molecular diagnostics	[24]

## Data Availability

No new data were created or analyzed in this study. Data sharing does not apply to this article.
